# Athletes’ Perceived Team Climate, Social Support, and Optimistic Thoughts During the COVID-19 Pandemic

**DOI:** 10.3390/ijerph22010046

**Published:** 2024-12-31

**Authors:** Chelsi E. Scott, Mary D. Fry, Troy O. Wineinger, Susumu Iwasaki, Haiying Long, Theresa C. Brown

**Affiliations:** 1 Department of Educational Psychology, School of Education, University of Kansas, Lawrence, KS 66045, USAtowineinger@ku.edu (T.O.W.); hlong@ku.edu (H.L.); 2 Department of Health and Human Performance, School of Arts and Sciences, Fort Lewis College, Durango, CO 81301, USA; siwasaki@fortlewis.edu; 3 School of Nursing and Health Studies, University of Missouri-Kansas City, Kansas City, MO 64110, USA; theresa.brown@umkc.edu

**Keywords:** collegiate sport, caring, ego-involving, task-involving, achievement goal perspective theory

## Abstract

In the Spring of 2020, Coronavirus 2019 (COVID-19) was officially declared a global pandemic, which prompted an unprecedented number of changes to societal functioning. Amongst those who experienced significant life alterations were collegiate athletes within the United States (US). The purpose of this study was to examine the relationship between US athletes’ perceptions of their team motivational climate, perceived support from coaches and teammates, and their optimistic thoughts during the COVID-19 pandemic. US collegiate athletes (N = 756; 56.3% female; Mage = 20.07 years, SDage *=* 1.57 years) across a variety of levels (e.g., Division I) and sports (e.g., basketball) were invited to participate in this study. Structural equation modeling analyses revealed significant positive associations between a caring and task-involving climate, athletes’ feeling supported by their coaches and teammates, and athletes’ optimistic thoughts during the COVID-19 pandemic. In addition, an ego-involving climate was significantly negatively associated with athletes’ feeling supported by their coaches and teammates. The final results suggest that the supportive actions of coaches and teammates during difficult times can mediate the positive connection between perceptions of a caring-task-involving climate on athletic teams and an athlete’s ability to stay optimistic during difficult life stressors.

## 1. Introduction

The coronavirus (COVID-19) was officially declared a pandemic in Spring 2020, promoting government-mandated prohibitions in the United States (US) on social gatherings. Individuals were encouraged to stay home and shelter in place, a decision that was particularly challenging for universities where students gather and live together in group settings. As universities closed campuses and transitioned to remote-learning, college students suffered. Students found that the abrupt transition to online learning, especially when learning resources were lacking (i.e., technology, internet access), was an added stressor to an already stressful situation [1]. Students during this time reported declines in daily behaviors that ultimately impacted their wellness and mood [2]. They also reported a loss of social support [3], resulting in isolation, loneliness, and fears of being diagnosed with COVID-19. These negative psychological consequences exacerbated their pre-existing mental health conditions, including psychological stress, anxiety, depression, and PTSD [1].

US college student athletes are a unique subset of college students, as US student athletes are expected to simultaneously excel in both the classroom and their sport [4]. As universities closed for the COVID-19 pandemic, many collegiate athletes were forced to end their competitive seasons, with safety protocols placing restrictions on competitions and practices. Athletes quickly lost access to accommodations afforded by their athletic contract with the university meant to help them excel, such as access to practice facilities, direct coach contact, social support of teammates, and a routine schedule. Many athletes reported that the pandemic disrupted their structured lifestyle, as training frequency and duration decreased. These changes negatively impacted athletes’ daily living, including decreases to their social interactions, physical activity levels, sleep patterns, and overall mental health, leading to increases in depression, feelings of sadness, a sense of loss, anxiety, and stress [5].

The COVID-19 pandemic lasted particularly long for US college athletics. As COVID-19 infection rates began to decrease, US athletic departments had to choose whether to allow in-person practice and competition and risk another outbreak or close for the season. For those schools choosing to allow competition, there was constant uncertainty of cancelled or postponed games and new regulations impacting how athletes could play their games (i.e., wearing masks, without fans, socially distant from teammates), all adding to college athletes’ stress. Stambulova et al. [6] discussed the unique challenges the pandemic presented for US college athletes, arguing that the uncertainty of COVID-19 effected all facets of their lives as they were growing into adulthood, including their athletic, psychological, psychosocial, academic, legal, and financial development.

Coaches and teammates were key factors within athletes’ support systems during this time. In a study financially supported by the National Collegiate Athletic Association (NCAA), Graupensperger and colleagues [7] found that over half of US college-level athletes reached out to their coaches and 90% reached out to their teammates on a weekly basis. Perceived social support and connectedness with teammates during the COVID-19 pandemic related to greater emotional, social, and psychological well-being among athletes. Interestingly, while athletes had more frequent contact with their peers and despite the positive findings when teammates stay connected, the study results suggested that coaches served as the primary social, mental, and athletic support during the COVID-19 pandemic.

### 1.1. Achievement Goal Perspective Theory

Given the important role that coaches and teammates play in athletes’ perceptions during difficulties such as the COVID-19 pandemic, it is important to identify the mechanisms that underlie their interactions. Achievement goal perspective theory (AGPT) [8] examines how athletes’ sport experiences can be optimized. According to Nicholls, coaches can create an ego-involving climate (EIC) or a task-involving climate (TIC) for their athletes. In an EIC, coaches (1) recognize and praise athletes’ ability and performance outcomes; (2) create and foster rivalry amongst teammates; (3) give most attention to the more talented athletes; and (4) punish mistakes [9]. In contrast, coaches in a TIC (1) value and recognize effort and improvement; (2) create and foster team cohesion; (3) ensure everyone has an important role on the team; and (4) treat mistakes as part of the learning process. An EIC is consistently related to negative psychological and emotional outcomes, including decreased motivation to achieve, greater anxiety, and an increase in negative affect, life stress, and physiological stress as measured by cortisol and inflammation responses [10,11,12]. Conversely, a TIC is associated with decreases in anxiety and life stressors and increases in motivation to achieve, positive coping strategies and affect, and effort and enjoyment within sports [12,13,14].

In addition to the beneficial features of a TIC, achievement motivation researchers have posited that a third component, termed the caring climate (CC), should be considered in tandem in sport settings, where athletes are genuinely cared for, valued, and treated with kindness and respect [15]. The benefits of perceiving a CC in sport and physical activities have been numerous, with athletes demonstrating greater emotional regulation, self-compassion, and the ability to adapt more quickly to difficulty in their sport when in CC [16]. When combined, athletes’ perceptions of a caring, task-involving (CTI) climate have been positively associated with their sport performance, including confidence to achieve, goal setting, mental preparation, and coachability [17]. In addition, a CTI climate has been positively related to broader life psychological outcomes, such as hope, happiness, and self-compassion, while negatively related to maladaptive outcomes, such as depressive symptoms, anxiety, fear, and stress [10,11,18].

In addition to the psychological benefits, a CTI climate may help facilitate positive life experiences beyond the sports realm. Specifically, athletes who perceive a CTI climate are more likely to consider their career plans once their athletic journey comes to an end [19]. Further, when coaches create a CTI climate, their athletes are more likely to indicate they would be honest in reporting concussion symptoms to their coach, even if their reporting would limit their playing time [20]. These findings suggest that, when athletes perceive their coaches to be caring and supportive, they are more likely to consider their long-term health and their after-sport lives, a finding especially salient during the COVID-19 pandemic.

### 1.2. Perceived Social Support

Reflective of the NCAA [4] findings of how many US collegiate athletes reached out to their teammates and coaches during the pandemic, researchers have found that a critical component for coping during challenging times is perceived social support, and indeed, coaches and teammates can be key to athletes’ social support networks [21,22,23,24]. Within sports, social support has been defined in many ways but consistently includes emotional and tangible support as primary tenants [21,24,25]. Emotional support has been operationalized as showing care and concern for athletes, attempting to understand and empathize with athletes’ experiences, and knowing when to avoid discussing negative events or performances [21]. Tangible support includes providing necessary resources for athletes to succeed, such as access to proper healthcare, academic tutoring, or other practical resources and assistance [21,25]. The benefits of athletes experiencing social support are numerous, including those centered around mental health. Specifically, perceived social support is negatively associated with symptoms of depression, sport helplessness, life stress, and burnout as well as positively associated with athletes’ resilience, gratitude, and sport satisfaction [25,26,27,28].

Further, researchers suggest that perceived social support may be even more critical during times of heightened stress or major life events. For example, athletes are more likely to incur an injury across the season when they have limited social support [29], and ironically, when they do experience a sports injury, athletes rely more heavily on coaches for social support [30]. Additionally, social support has also been found to buffer the risk of burnout during high-stress periods [27]. One way to help facilitate perceived social support among athletes is through creating a CTI climate. A CTI climate is positively associated with the social support subscale of the Leadership Scale for Sports among adolescent athletes [31] and with perceptions of teacher support in collegiate physical activity classes [32]. It follows that, due to the nature of a CTI climate, creating an environment in which athletes feel cared for and supported, valued, and where team cohesion is fostered and encouraged, athletes would be more likely to report greater feelings of social support from coaches and teammates during COVID-19.

### 1.3. Optimism

Within clinical psychological research, optimism is often conceptualized as a trait that remains relatively stable and reflects an individual’s belief that, in general, there will be positive things that occur to them in the future [33]. Optimism has been linked to greater mental and physical health as well as quality of life [34]. Further, optimism is associated with greater coping mechanisms as well as greater engagement in more proactive goal pursuits [33]. Within the sport psychology literature, optimism has been negatively related to stress and burnout among competitive athletes [35]. More recently, optimism was found to mediate the relationship between athletes’ social support and their psychological well-being [36]. However, to date, there is little research examining perceived motivational climates with optimism, especially during unique circumstances such as the COVID-19 pandemic. The pandemic offered a unique opportunity to study individuals’ optimism during a very challenging period, a perspective that is not necessarily captured with current measures of optimism.

The purpose of this study was to examine the connection between perceptions of the climate (CC, TIC, and EIC) on team sports, perceived coach and teammate support, and optimistic thoughts (OT). Specifically, the research team was interested in whether perceived support from coaches (SFC) and support from teammates (SFT) mediated the relationship between athletes’ perceptions of the motivational climate on their college team and their OT during the COVID-19 pandemic. It was hypothesized that, when athletes perceived a CC and TIC on their teams, they would also perceive greater SFC and SFT and report more OT during the COVID-19 pandemic. In contrast, athletes who perceived an EIC would perceive less SFC and SFT and report less OT during the COVID-19 pandemic.

## 2. Materials and Method

### 2.1. Participants

During Spring 2020 after the World Health Association declared COVID-19 a pandemic, the research team invited collegiate athletic departments across the US to share an online survey with their student athletes via Qualtrics (N = 756; Mage = 20.07 years; SDage = 1.57 years; 56.3% female; 43% male; 0.7% non-binary/other). Participation in this study was voluntary, and the athletes were informed that their responses would be confidential. The athletes identified as White (66.6%), Hispanic or Latino (10.9%), or another race/ethnicity (<10%). The athletes represented each school classification, including freshman (30.2%), sophomore (23.6%), junior (27.5%), senior (14.3%), and fifth-year (2.6%) undergraduate students. In addition, graduate students (1.8%) were represented in the sample. The athletes indicated receiving a partial scholarship (50%), no scholarship (40.3%), and full scholarship (9.7%). All university divisions were represented in the sample, including Division I (8.7%), Division II (27.8%), Division III (39.5%), NAIA (21%), and other (3%). Permission to conduct this study was obtained from the lead researcher’s institutional review board.

### 2.2. Measures

The athletes were asked to respond to questions with respect to their current collegiate team. Total scores for each scale were calculated by averaging the participants’ responses.

#### 2.2.1. Caring Climate Scale

Given the quick turn-around time to produce a survey when universities closed for the pandemic and knowing that long surveys would likely result in fewer responses, an abbreviated version of the 13-item Caring Climate Scale was created (CCS) [15]. Five items were chosen based on factor loadings from previous research that strongly represented the features of each climate and demonstrated internal reliability for the CCS with collegiate athletes [17]. Furthermore, a group of motivational researchers consisting of faculty and graduate students reviewed and discussed the chosen items. The abbreviated 5-item CCS included the stem “On this team”, and the scale assessed the extent to which the athletes perceived their team environments to be welcoming and where team members were treated with kindness and respect. A sample item is “On this team, the coach cares about the athletes”, and the athletes responded to the items on a 5-point Likert scale ranging from 1 (strongly disagree) to 5 (strongly agree).

#### 2.2.2. Perceived Motivational Climate in Sport Questionnaire

Similar to the CCS, an abbreviated version of the 21-item Perceived Motivational Climate in Sport Questionnaire (PMCSQ) [37] was created to examine the athletes’ perceptions of the TIC (5 items) and EIC (5 items) climates on their teams. While the PMCSQ does not have an abbreviated version, Moore et al. [38] demonstrated reliability for an abbreviated 12-item version of the Perceived Motivational Climate in Exercise Questionnaire (PMSEQ), a scale that was originally adapted from the PMCSQ. Furthermore, and similar to the process used for the CCS, factor loadings from previous research [17] were examined to choose the 10 items to include in the current study, and a team of faculty and graduate students reviewed and discussed before finalizing. The stem for each item for the current abbreviated PMCSQ was “On this team ...”. A sample for the TIC sub-scale is “On this team, trying hard is rewarded”, and a sample for the EIC sub-scale is “On this team, doing better than others is important”. The participants responded to the items using a 5-point Likert scale ranging from 1 (strongly disagree) to 5 (strongly agree).

#### 2.2.3. Perceived Support from Coach(es) (SFC)

For this study, the research team developed a 5-item measure to assess the degree to which athletes perceived support from their coach during the COVID-19 pandemic. These items were reviewed and discussed by faculty and graduate students in sport psychology. A sample item is “My coach has checked in with me often to be sure I’m doing okay”. The athletes were asked to respond on a 5-point Likert scale ranging from 1 (strongly disagree) to 5 (strongly agree). Internal reliability and factor loadings were included in the analysis for this study.

#### 2.2.4. Perceived Support from Teammates (SFT)

For this study, the research team developed a 4-item measure to assess the extent to which athletes felt they received support from their teammates during the COVID-19 pandemic. These items were reviewed and discussed by faculty and graduate students in sport psychology. A sample item from this measure is “My teammates have helped me deal with the disappointment of our season/semester ending”. The athletes were asked to respond on a 5-point Likert scale ranging from 1 (strongly disagree) to 5 (strongly agree). Internal reliability and factor loadings were included in the analysis for this study.

#### 2.2.5. Optimistic Thoughts During the COVID-19 Pandemic (OT)

For purposes of this study, a 5-item measure was created to assess the level of optimism the athletes were experiencing during the COVID-19 pandemic. These items were reviewed and discussed by faculty and graduate students in sport psychology. A sample item is “I’m confident I’ll come out of this pandemic emotionally and physically strong”. The athletes were asked to respond on a 5-point Likert scale ranging from 1 (strongly disagree) to 5 (strongly agree). Internal reliability and factor loadings were included in the analysis for this study.

### 2.3. Statistical Analyses

Preliminary data examination and analyses were conducted in IBM SPSS version 29 [39]. All the variables were normally distributed. Missing values in the dataset were analyzed by using Little’s MCAR (Missing Completely at Random) test. The result was not significant, indicating the values were missing completely at random (χ^2^ _(246)_ = 230.08, *p* = 0.76).

After the preliminary data examination, all analyses were performed in *Mplus* 8.11, a statistical modeling software program that provides flexible tools to analyze latent variable models [40]. As a first step, confirmatory factor analysis (CFA) was conducted to assess the measurement model and support construct validity of the measures [41]. Then, latent variable parallel mediator mediation analysis was used to test the research hypotheses through the structural equation modeling (SEM) approach, which can consider measurement errors and provides an unbiased estimate of multiple mediation effects while examining the structural relationships among variables [42,43,44].

Given the high correlations among the three climate variables, we analyzed three separate mediation models under the SEM framework, with each climate variable being the only exogenous variable in the model. Previous studies that have found CTI climates to be highly correlated with each other and negatively correlated with EIC have consistently demonstrated adequate reliabilities on the CC, TIC, and EIC variables (e.g., [12,45]). Specifically for the current study, we considered Model 1: CC—SFC and SFT—OT; Model 2: TIC—SFC and SFT—OT; and Model 3: EIC—SFC and SFT—OT. This approach allowed for us to best distinguish the contribution of each component of the motivational climate to the overall results and examine the unique direct and indirect effects of each climate variable (CC, TIC, EIC) on the two mediators (SFC and SFT) and endogenous variable (OT). It is also in line with previous motivational climate research considering mediation models [45]. In addition, we accounted for the error variance of SFC and SFT in the models. Scholarship (SCH), measured on a scale of 1 (no scholarship) to 3 (full scholarship), was included as a control variable and tested for its direct effect on OT, as having funding to attend school could be a differentiator in athletes thinking optimistically during the pandemic.

The model fit indices used in interpreting the results include the Comparative Fit Index (CFI), Tucker–Lewis Index (TLI), Root Mean Square Error of Approximation (RMSEA), and Standardized Root Mean Square Residual (SRMR). A model is often considered good if the CFI and TLI are 0.90 or above, RMSEA is 0.06 or below, and SRMR is 0.08 or below [46,47]. *Mplus* uses the *STDYX* function to produce standardized coefficients of the variables and uses the full information maximum likelihood (FIML) approach to handle missing values, which has yielded unbiased results. Although many methods have been proposed to test indirect or mediated effects during the past few decades, such as traditional Baron and Kenny’s causal-step test [48], Sobel’s test [49], bootstrap test for the confidence interval (CI) [50], bias-corrected bootstrap test for CI [51], and Bayesian mediation method [52], the bootstrap CI method (with 1000 bootstraps) was selected in this study because it can control Type I error while producing accurate values [53].

## 3. Results

### 3.1. Descriptive Statistics

Descriptive statistics (means, SDs, and actual composite score ranges for each scale), Cronbach’s alphas, and zero-order correlations are presented in Table 1. The alphas for all the scales were satisfactory. The three exogenous variables (CC, TIC, EIC) and two parallel mediators (SFC, SFT) were all significantly correlated (*p* < 0.001).

### 3.2. Confirmatory Factor Analysis

**Figure 1:**
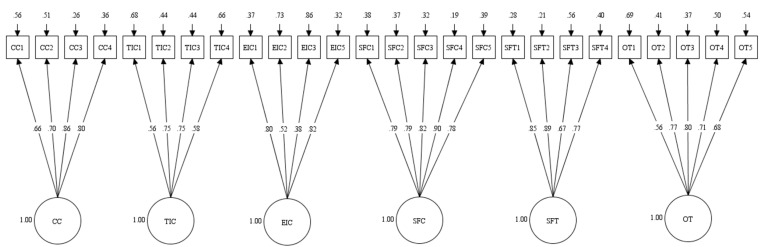
Confirmatory Factor Analysis. Note. CC = Caring Climate; TIC = Task-Involving Climate; EIC = Ego-Involving Climate; SFC = Support from Coaches; SFT = Support from Teammates; OT = Optimistic Thinking.

The original measurement model, including the measures of CC, TIC, EIC, SFC, SFT, and OT, was examined by using a CFA, resulting in an adequate model fit (CFI = 0.93, TLI = 0.92, SRMR = 0.057, RMSEA = 0.051, 90% CI [0.047–0.054]). Most factor loadings were strong, but three items (CC5, TIC5, and EIC4) were lower than 0.40; thus, they were removed from the model. This change yielded a much better model fit and was used for all models going forward (CFI = 0.96, TLI = 0.95, SRMR = 0.040, RMSEA = 0.040, 90% CI [0.037–0.046])(Figure 1). The removal of CC5 and TIC 5 increased the Cronbach’s alphas of the CC and TIC measures to 0.84 and 0.76, respectively, while the removal of EIC4 yielded an identical (0.72) Cronbach’s alpha of EIC measures.

### 3.3. Mediation Analysis

#### 3.3.1. Model 1 (CC Model: CC– SFC and SFT– OT)

**Figure 2:**
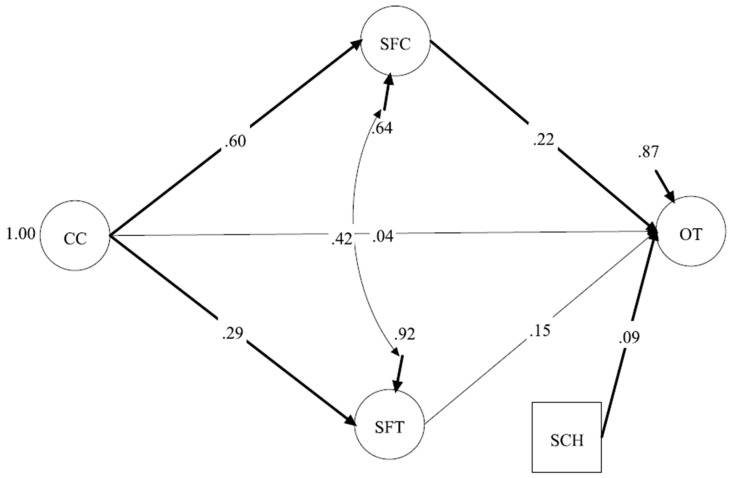
Model 1: CC—SFC and SFT—OT. Note. TIC = Task-Involving Climate; SFC = Support from Coaches; SFT = Support from Teammates; OT = Optimistic Thinking; SCH = Scholarship. All significant relationships are in bold. Observed variables and factor loadings are excluded from the figure for space; however, factor loadings are very close to those in the CFA.

The first SEM model was conducted with CC as the only exogenous variable (Figure 2). The results demonstrated very good model fit (CFI = 0.97, TLI = 0.96, SRMR = 0.038, RMSEA = 0.046, 90% CI [0.040–0.053]). CC did not have a significant relationship with OT (β = 0.04, *p* = 0.62), but it had significant positive direct effects on SFC (β = 0.60, *p* < 0.001) and SFT (β = 0.29, *p* < 0.001) and significant positive indirect effects on OT through either SFC (β = 0.14, *p* = 0.002, 97.5% CI [0.047, 0.226]) or SFT (β = 0.04, *p* = 0.021, 97.5% CI [0.009, 0.080]). Additionally, SFC (β = 0.22, *p* = 0.002) and SFT (β = 0.15, *p* = 0.014) both had significant positive effects on OT. SCH also had a significant relationship with OT (β = 0.09, *p* = 0.03) (Figure 2). These results indicate that the relationship between CC and OT was partially mediated by SFC and SFT. The model explained 36.5% of the variance in SFC, 8.4% of the variance in SFT, and 12.7% of the variance in OT.

#### 3.3.2. Model 2 (TIC Model: TIC—SFC and SFT—OT)

**Figure 3:**
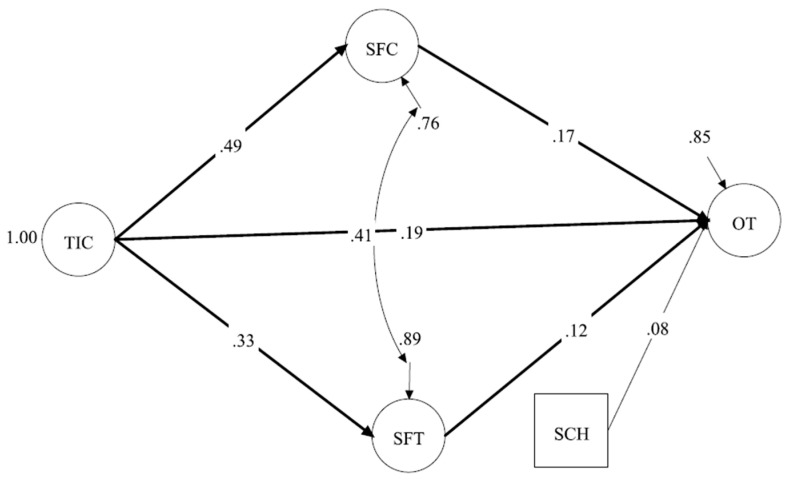
Model 2: TIC—SFC and SFT—OT. Note. TIC = Task-Involving Climate; SFC = Support from Coaches; SFT = Support from Teammates; OT = Optimistic Thinking; SCH = Scholarship. All significant relationships are in bold. Observed variables and factor loadings are excluded from the figure for space; however, factor loadings are very close to those in the CFA.

A second SEM model was conducted with TIC as the only exogenous variable (Figure 3). The results demonstrated a very good model fit (CFI = 0.97, TLI = 0.96, SRMR = 0.039, RMSEA = 0.044, 90% CI [0.037–0.050]). TIC had a significant positive relationship with SFC (*β* = 0.49, *p* < 0.001), SFT (*β* = 0.33, *p* < 0.001), and OT (*β* = 0.19, *p* = 0.002). TIC also had significant positive indirect effects on OT through SFC (*β* = 0.08, *p* = 0.010, 97.5% CI [0.022, 0.147]) or through SFT (*β* = 0.04, *p* = 0.047, 97.5% CI [0.001, 0.083]). Both SFC (*β* = 0.17, *p* = 0.007) and SFT (*β* = 0.12, *p* = 0.042) had significant positive relationships with OT. SCH had a marginally significant effect on OT (*β* = 0.08, *p* = 0.052) (Table 2). These results suggest that, when athletes perceived a TIC, they were more likely to feel SFC, SFT, and OT. SFC and SFT partially mediated the relationship between perceptions of the TIC and OT. The model explained 23.7% of the variance in SFC, 11.2% of the variance in SFT, and 15.1% of the variance in OT.

#### 3.3.3. Model 3 (EIC Model: EIC– SFC and SFT—OT)

**Figure 4:**
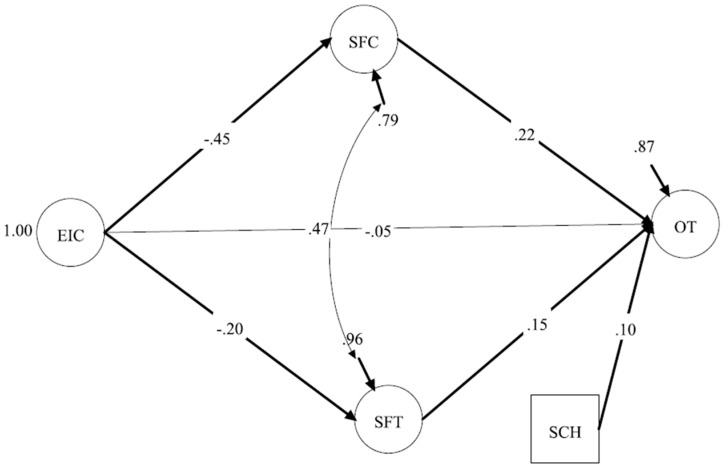
Model 3: EIC—SFC and SFT—OT. Note. EIC = Ego-Involving Climate; SFC = Support from Coaches; SFT = Support from Teammates; OT = Optimistic Thinking; SCH = Scholarship. All significant relationships are in bold. Observed variables and factor loadings are excluded from the figure for space; however, factor loadings are very close to those in the CFA.

A final SEM model was conducted with only EIC as the exogenous variable (Figure 4). The results demonstrated a very good model fit (CFI = 0.97, TLI = 0.96, SRMR = 0.039, RMSEA = 0.045, 90% CI [0.039–0.051]). The results showed that EIC did not have a significant relationship with OT (*β* = −0.05, *p* = 0.387) but had significant negative direct effects on SFC (*β* = −0.45, *p* < 0.001) and SFT (*β* = −0.20, *p* < 0.001) and significant indirect effects on OT through SFC (*β* = −0.10, *p* = 0.001, 97.5% CI [−0.166, −0.046]) and SFT (*β* = −0.03, *p* = 0.032, 97.5% CI [−0.058, −0.006]). SFC (*β* = 0.22, *p* < 0.001) and SFT (*β* = 0.15, *p* = 0.012) still had significant positive relationships with OT. SCH also had a significant positive relationship with OT (*β* = 0.10, *p* = 0.021) (Table 2). These results suggest that those athletes who perceived an EIC were not likely to have OT and were also less likely to perceive SFC or SFT. The model explained 20.6% of the variance in SFC, 3.9% of the variance in SFT, and 12.8% of the variance in OT.

## 4. Discussion

The purpose of this study was to examine the experiences college athletes had during the COVID-19 pandemic. Specifically, perceived SFC and SFT were expected to mediate the relationship between athletes’ perceptions of the motivational climate on their college team and their OT during the COVID-19 pandemic. Partial support was found for the hypothesis, with SFC partially mediating a positive relationship between a CC, TIC, and OT. The connection between a supportive coach who is available during times of adversity and perceptions of a high CTI climate is in line with AGPT and CC research, as coaches play a critical role in influencing perceptions of the motivational climate in sport settings [54]. Likewise, SFT partially mediated a positive relationship between TIC and OT. Cooperation among teammates and believing that everyone plays an important role are hallmarks of a TIC [8], and these concepts are in line with teammates showing support during difficult times.

SFC and SFT partially mediated a negative relationship between EIC and OT, although SFC and SFT were positively directly related to OT. The ability to still have OT is perhaps not surprising in these results given that the ego-involving aspects of the team were likely less pronounced during the pandemic since many athletes were dispersed from dorms back to their hometowns, teams were unable to practice and compete, and coaches and athletes spent much less time together. As such, it is conceivable that coaches and teammates could still rally in times of adversity such as a pandemic, show support for athletes, and show a direct association with OT. Regardless, when taken together in the full EIC model, athletes who perceived an EIC were less likely to perceive SFC or SFT and less likely to have OT, a finding reflective of previous research showing a connection between EIC and maladaptive emotional and physiological responses to stressful events [10,12].

### 4.1. Motivational Climate Impact

When considered individually, the CC and the TIC were each positively associated with feeling support from the coaches and teammates, while the EIC was negatively associated with those feelings of support. In addition, the CTI climates were associated with OT. These results align with AGPT and CC theoretical tenants that highlight the important role that coaches and teammates play in helping athletes feel supported, welcomed, and a valued part of the team [8,15,54] and with previous research linking social support in athletic endeavors with mental health benefits [7,25,27,28]. Overall, the results of this current study extend previous AGPT and CC research, suggesting that, when coaches and teammates offer support and make themselves available to athletes, these actions can influence perceptions of the climate, optimize athletes’ experiences, and even impact mental well-being [10,11,17,18,54,55].

While this study considered the climates individually, in practical terms, researchers advocate for emphasizing both a CC and TIC in tandem to yield the most adaptive responses [12,17,20]. Within sports, researchers have found that, when athletes perceive a CTI climate, they not only feel more support from their coaches and teammates but also show more support to others [56]. The current results suggest that, by demonstrating their support, coaches and teammates can embody the aspects of a CTI climate by specifically demonstrating mutual kindness and respect and engaging in actions that validate that everyone plays an important role. Further, perceptions of a CTI climate have been associated with greater emotional, psychological, and physical health [12,17,18]. It follows that, by being available to support one another, the coaches’ and teammates’ support would act as a connector between perceptions of the CTI climate and the ability to focus on OT during a challenging time.

### 4.2. Motivational Climate and Optimistic Thoughts

The skill of being able to think optimistically during times of adversity and stress can be so beneficial to a person’s well-being that experts in psychology advocate for understanding the antecedents that can lead to OT [57]. The present study suggests there is a relationship between cultivating perceptions of the CTI climate in team settings and student athletes’ abilities to utilize optimistic thinking during unforeseen and unprecedented events. These findings could have important implications for practitioners interested in helping athletes utilize optimism as a coping tool when difficulties arise. During the initial stages of the COVID-19 pandemic, changes to life routines happened quickly. College athletes experienced abrupt ends to their seasons, rapid separation from support networks, and a loss of training facility access. Many athletes reported feeling isolated and disconnected from their coaches and teammates, with these significant lifestyle disruptions influencing their physical, psychological, and mental well-being [5]. Coping tools work best when individuals work on them during non-stressful times. Researchers have found that, when athletes work on strengthening their coping mechanisms, they experience greater mental health and less anxiety than those who do not receive similar training [58]. OT is a coping skill that could be strengthened and developed throughout the collegiate sport experience by creating and cultivating CTI climates.

### 4.3. Support from Coaches and Peers as Mediators

The current research found that SFC plays a significant role in influencing the relationship between perceptions of the climate and whether athletes can stay optimistic during challenging times. This finding is important to those in coaching roles, as athletes listed their coaches as their top form of social support, relying primarily on their coaches as their athletic support during the COVID-19 pandemic [4]. Coaches’ interactions, expectations, definitions of excellence, and efforts to build community all contribute to athletes’ perceptions of the motivational climate during their athletic experiences [55]. Those coaches who intentionally foster CTI climates positively impact athletes’ development beyond the specific sport, including helping athletes to develop self-compassion and emotional regulation skills that contribute to a positive life and are particularly useful to cope in times of adversity [16]. Being able to focus on optimistic thoughts is a life skill that serves a far greater purpose than just during pandemics; individuals who can rely on optimism and positive thinking during times of adversity report less stress, negative emotions, and depressive symptoms and greater immune system functioning, vitality, quality of life, and health-related outcomes [59].

Though SFC was the greater contributor within each model, SFT was still a significant contributor within the current findings. In all three models, perceptions of the climate were associated with SFT, a positive association for CC and TIC, and a negative association for EIC. Researchers interested in the motivational climate discuss the important role that teammates, not just coaches, play in fostering perceptions. Nicholls and Hazzard [60] discuss the challenges of fostering strong relationships among all members of a group and that this is an important component when cultivating a CTI climate. Likewise, the educational philosopher Noddings [61] discussed the reciprocal nature of the CC, suggesting that both teacher and student must be involved for caring to take place. The current results demonstrate that perceptions of the climate and SFT are related, and coaches might consider ways to involve interaction among all members of the team when working to establish a CTI climate. Even during adverse times such as the COVID-19 pandemic, it is possible for coaches to find unique ways to stay engaged with their teams, helping athletes connect, create, and maintain strong ties [18], and it is reasonable to assume that, as athletes echo these efforts, the psychological and mental benefits are even more powerful.

### 4.4. Limitations

While the benefits of this study are numerous, there are several limitations to note. First, multiple data collections at different time points would have been ideal to understand how the athletes were coping over time as the COVID-19 pandemic continued. With a single data point, the current study provides a limited picture of the athletes’ experiences, with causality and directionality not determined. Research has shown that the longitudinal benefits of a CTI climate are numerous, and having multiple timepoints could have provided an important view of how a CTI climate provides social support and subsequently aids in maintaining an optimistic outlook across the COVID-19 pandemic. It would also have allowed for examining how the athletes’ perceptions of SFC and SFT have changed throughout the COVID-19 pandemic.

Secondly, some of the measures were abbreviated, and using the full, validated measure may have provided additional results. The researchers made the decision to abbreviate some of the measures to move swiftly and nimbly during an unprecedented, quickly evolving situation. When the pandemic reached full force, there was a limited window to develop the surveys, obtain IRB approval, and distribute to student athletes. The truncated survey items allowed for a quicker distribution of the survey to capture the athletes’ perceptions of their team climate while the climate was still salient in their minds. Being mindful of presenting the athletes with a brief survey was also important to ensure a higher response rate. Should future researchers find themselves in a similar predicament, it should be noted that this research team was still thoughtful and careful, choosing items that demonstrated high factor loadings in previous research and in consultation with sport psychology experts, coaches, and current athletes. Additionally, by employing a CFA, the research team was able to discard the few items that did not load well, resulting in good indicators for the perceived motivational climate, perceived SFC and SFT, and experiencing OT during the pandemic. Ideally though, future researchers will employ the full measures. In addition to making the decision to abbreviate some previously validated surveys, the team had to create some survey measures specifically for this study.

Third, the OT survey items were developed specifically for student athletes who perceived the pandemic as a stressful event on both their sport and life. However, the study sample included athletes from different US college divisions, sports, and geographical locations whose sport and life circumstances could have been very different. For example, student athletes competing in winter and spring sport seasons may have received another year of eligibility. For some, this could be a positive outcome as they were given another opportunity to train and excel at their sport. For others, another year might have conflicted with other life goals they had, resulting in a stressful decision. Likewise, whether student athletes had funding in the form of a scholarship did explain some of the variability in OT, suggesting that the level of worry the athletes may have experienced was variable depending on life circumstances. How the participants interpreted the outcomes caused by the COVID-19 pandemic could have influenced how they responded to the OT measure.

Finally, this study included a variety of different athletes from various backgrounds, sports, and institutional levels. It may have been beneficial to target one specific group (e.g., sport, Division I) to investigate rather than examine the experiences of all athletes, as doing so may have resulted in different findings. Given the unknowns of the COVID-19 pandemic and the opportunity to sample a large population, the research team opted to include as many student athletes as possible. As a result, the present study represents one of the largest sample sizes in CTI climate research with collegiate athletes.

### 4.5. Future Direction

While the 2020 US government restrictions for the COVID-19 pandemic have ended, university and sport programs continue to face challenges. Future researchers could follow sport teams’ transition back to play as well as continue to investigate the long-term impact and longevity of skills that are associated with CTI climates, such as OT. Likewise, continued research to understand the supportive behaviors that coaches and teammates engage in that help mediate the positive relationship between CTI climates and adaptive life skills will further contribute to motivational climate literature and speak to the benefits of coaches creating a CTI climate on their teams during adverse events.

Another direction for future research would be to explore the benefits of a CTI climate for those who may be at an even greater risk for mental health concerns, especially given the events that took place during the COVID-19 pandemic. Historically oppressed groups, such as LGBTQ+, women, and athletes of color, are at a greater risk of mental health difficulties and in greater need of mental health services than their respective counterparts [62,63,64]. This is an area that is open to future study, and already, the results are promising. Claunch and colleagues [65] found that retention rates dramatically improved among a group of indigenous collegiate athletes when their coaching staff were trained to create a CTI climate. While the present study provides evidence that a CTI climate is beneficial for all athletes, examining the specific benefits to these groups may result in a better understanding of the unique challenges and potential solutions for these marginalized populations. Fry et al. [54] published a training manual for coaches and athletes that provides strategies they may consider that foster a CTI climate in sport and physical activity settings.

Finally, while the focus of the present study was examining student athlete experiences during COVID-19, there may be some implications for coaches as well. Coaches may experience positive self-outcomes when they recognize how their actions favorably impact their athletes’ psychosocial development [66]. Moreover, researchers could investigate how coaches’ efforts to create and maintain a CTI climate during extreme adverse situations, such as the COVID-19 pandemic, help athletes reframe how they experience events and how these experiences could extend beyond their athletic careers.

Coaches may also benefit from these reframing abilities, not just in their interactions with athletes but also in their personal lives. The impact on coaches is a promising area for future research.

## 5. Conclusions

Overall, the present study contributes to the current benefits of a CTI climate among collegiate athletes, specifically investigating the link between perceptions of the climate and the ability to have optimistic thoughts during extreme adverse events such as a global pandemic. The COVID-19 pandemic presented challenges for athletes, threatening their physical, emotional, and psychological health, presenting a unique opportunity to better understand how supportive behaviors from coaches and teammates might explain the relationship between perceptions of the climate on a team sport and the ability to utilize optimistic thinking during difficulties. Creating a CTI climate for athletic teams may be one way that coaches can help their athletes mitigate the effects of life stressors, especially when both the coaching staff and teammates give their support, which can act as a potential connector between perceptions of climate and the ability to think optimistically when times are difficult. In doing so, student athletes may experience significant benefits to their emotional and psychosocial health.

## Figures and Tables

**Table 1:** Means, standard deviations (SD), minimum, maximum, reliability (α), and correlations for all athletes.

Measure	M	SD	Min	Max	α	1	2	3	4	5
1. CC	4.24	0.58	1.80	5.00	0.79					
2. TIC	4.17	0.62	1.60	5.00	0.75	0.68 ***				
3. EIC	3.37	0.72	1.20	5.00	0.73	−0.46 ***	−0.42 ***			
4. SFC	3.64	0.93	1.00	5.00	0.91	0.54 ***	0.44 ***	−0.34 ***		
5. SFT	3.53	0.87	1.00	5.00	0.87	0.38 ***	0.33 ***	−0.19 ***	0.46 ***	
6. OT	3.84	0.72	1.00	5.00	0.82	0.24 ***	0.28 ***	−0.12 ***	0.29 ***	0.26 ***

Note. CC = Caring Climate, TIC = Task-Involving Climate, EIC = Ego-Involving Climate, SFC = Support from Coaches, SFT = Support from Teammates, OT = Optimistic Thoughts. Note. All measures used a 1 to 5 Likert scale. *** *p* < 0.001.

**Table 2:** Standardized coefficient, standard error, confidence interval of direct and indirect effects in CC, TIC, and EIC models.

		CC Model		
**Direct Effects**	**SCoefficient**		**S.E.**	***p* Value**
CC 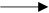 SFC	0.604 **		0.035	0.000
CC 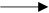 SFT	0.291 **		0.045	0.000
CC 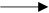 OT	0.036		0.071	0.615
SFC 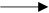 OT	0.223 *		0.071	0.002
SFT 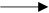 OT	0.147 *		0.059	0.014
SCH 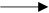 OT	0.093 *		0.042	0.026
**Indirect Effects**	**SCoefficient**	**97.5% CI**	**S.E.**	***p* value**
CC 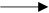 SFC 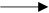 OT	0.135 *	0.047–0.226	0.045	0.002
CC 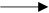 SFT 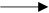 OT	0.043 *	0.009–0.080	0.018	0.021
		**TIC Model**		
**Direct Effects**	**SCoefficient**		**S.E.**	***p* value**
TIC 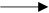 SFC	0.486 **		0.045	0.000
TIC 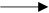 SFT	0.334 **		0.047	0.000
TIC 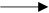 OT	0.189 *		0.062	0.001
SFC 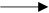 OT	0.166 *		0.061	0.007
SFT 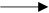 OT	0.122 *		0.060	0.042
SCH 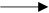 OT	0.081		0.042	0.052
**Indirect Effects**	**SCoefficient**	**97.5% CI**	**S.E.**	***p* value**
TIC 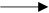 SFC 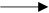 OT	0.081 *	0.022–0.147	0.031	0.010
TIC 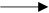 SFT 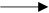 OT	0.041 *	0.001–0.083	0.021	0.047
		**EIC Model**		
**Direct Effects**	**SCoefficient**		**S.E.**	***p* value**
EIC 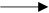 SFC	−0.454 **		0.040	0.000
EIC 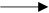 SFT	−0.198 **		0.046	0.000
EIC 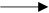 OT	−0.048		0.056	0.387
SFC 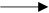 OT	0.221 *		0.063	0.000
SFT 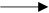 OT	0.149 *		0.059	0.012
SCH 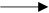 OT	0.096 *		0.042	0.021
**Indirect Effects**	**SCoefficient**	**97.5% CI**	**S.E.**	***p* value**
EIC 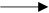 SFC 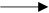 OT	−0.100 **	−0.166–−0.046	0.029	0.000
EIC 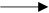 SFT 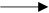 OT	−0.030 *	−0.058–−0.006	0.014	0.032

Note. CC = Caring Climate; TIC = Task-Involving Climate; EIC = Ego-Involving Climate; SFC = Support from Coaches; SFT = Support from Teammates; OT = Optimistic Thinking; SCH = Scholarship; SCoefficient = Standardized Coefficient; CI = Confidence Interval; S.E. = Standard Error. Arrows in the direct and indirect effects show the directions of the relationships. * *p* < 0.05; ** *p* < 0.001.

## Data Availability

The datasets presented in this article are not readily available because of ethical restrictions by the IRB. Requests to access the datasets should be directed to the corresponding author.
